# Molecular Survey and Genetic Analysis of *Ehrlichia canis* in *Rhipicephalus sanguineus* Ticks Infesting Dogs in Northern Taiwan

**DOI:** 10.3390/microorganisms13061372

**Published:** 2025-06-12

**Authors:** Chien-Ming Shih, Pei-Yin Ko, Li-Lian Chao

**Affiliations:** 1 Graduate Institute of Medicine, College of Medicine, Kaohsiung Medical University, Kaohsiung 807, Taiwan; cmshih@kmu.edu.tw; 2 Graduate Institute of Pathology and Parasitology, National Defense Medical Center, Taipei 114, Taiwan; 3 Department of Medical Research, Kaohsiung Medical University Hospital, Kaohsiung 807, Taiwan

**Keywords:** *Ehrlichia canis*, zoonosis, *Rhipicephalus sanguineus*, tick, Taiwan

## Abstract

*Ehrlichia canis* is a tick-transmitted zoonotic pathogen in dogs. We conducted a molecular survey for screening of *E. canis* infection in *Rhipicephalus sanguineus* ticks infesting dogs and identified its genetic identity in Taiwan. A total of 1195 *R. sanguineus* ticks were collected and examined for *Ehrlichia* infection by nested-PCR assay targeting the 16S ribosomal RNA (rRNA) gene. In general, *Ehrlichia* infection was detected in 1.5% of examined ticks, and was detected in nymph, male and female stages with infection rates of 0.6%, 1.31% and 2.76%, respectively. The highest monthly prevalence was observed in August with an infection rate of 5.91%. Genetic identity was analyzed by comparing the 16S rRNA gene sequences obtained from 11 Taiwan strains and 15 other strains representing five genospecies of *Ehrlichia* spp., including two outgroups (*Anaplasma phagocytophilum* and *Rickettsia rickettsii*). Results revealed that all Taiwan strains were genetically affiliated to the same clade within various *E. canis* strains documented in GenBank with a high sequence similarity (99.7–100%) and that they can be clearly distinguished from other genospecies of *Ehrlichia*. This study provides the first evidence of *E. canis* identified in *R. sanguineus* ticks and highlights the potential threat for human infections in Taiwan.

## 1. Introduction

The genus *Ehrlichia* includes five species of gram-negative obligate intracellular bacteria infecting monocytes, and *E. canis* is recognized as the primary causative agent for canine monocytic ehrlichiosis (CME), a potentially fatal disease in dogs [1,2]. This *Ehrlichia* infection was described for the first time in Algeria in 1935 [3], and it has been reported in the southern regions of the USA [4] and was recorded in some western and southern regions of Europe [5]. Currently, this infection is widely distributed around the world, and the prevalence of *E. canis* depends on the distribution of its vector ticks. Although *E. canis* had been reported in asymptomatic dogs of central Taiwan [6], there is no confirming evidence for the existence of *E. canis* in its vector tick. Thus, a molecular survey on tick-borne *E. canis* in *Rhipicephalus sanguineus* ticks is crucial to understand the potential threat of emerging tick-borne *E. canis* infections in Taiwan.

The *R. sanguineus* tick is a haematophagous arthropod and is commonly parasitic on canine hosts throughout the world [7,8]. Previous studies revealed that *R. sanguineus* has been recognized as the major vector for the transmission of various tick-borne zoonotic pathogens, such as *Babesia*, *Ehrlichia*, *Anaplasma*, *Hepatozoon* and *Rickettsia*, among animals and humans [9,10,11]. Because of the increasing detection of *H. canis*, *B. vogeli*, *B. gibsoni*, *A. platys* and various *Rickettsia* spp. in *R. sanguineus* ticks of Taiwan [12,13,14,15,16,17], this tick species has become the focus of research on medical and veterinary importance. Although the *R. sanguineus* ticks had been recognized as the incriminated vector ticks for a variety of zoonotic pathogens, there has been no investigation confirming the prevalence and genetic identity of *E. canis*, a zoonotic pathogen for human infection, in this tick species in Taiwan.

Molecular detection targeting the 16S rRNA gene of *Ehrlichia* has made the feasibility for identifying the genetic identity within the vector ticks. Indeed, this molecular tool can be used to differentiate the genetic variance at the individual base-pair level and gives a direct method for measuring the genetic diversity between and within species of *Ehrlichia* [18,19]. Previous studies based on the molecular marker of 16S rRNA genes of *Ehrlichia* have demonstrated that it is sufficiently informative for the genetic analysis of phylogenetic relationships between the genetic diversity of *Ehrlichia* species among various vectors and hosts [20,21,22,23,24,25,26]. Thus, molecular detection and genetic analysis based on the genetic comparison of 16S rRNA genes have made it feasible to facilitate the identification and discrimination of *Ehrlichia* species within ticks.

The objectives of this study are to investigate the prevalence of *Ehrlichia* infection in *R. sanguineus* ticks collected from Taiwan and to determine the genetic identity of *Ehrlichia* spp. in *R. sanguineus* ticks. The genetic affiliation of *Ehrlichia* strains detected in *R. sanguineus* ticks of Taiwan was analyzed by comparing their nucleotide composition with other *Ehrlichia* strains identified from various biological origins and geographical sources documented in GenBank.

## 2. Materials and Methods

### 2.1. Tick Collection and Species Identification

A total of 1195 *R. sanguineus* ticks used in this study were collected from 236 dogs in twelve districts of Taipei City in northern Taiwan, and only 3 districts (Shihlin, Wanhua and Sinyi) were detected with positive ticks. All these dogs were handled by a veterinary practitioner for collecting the attached ticks. All these ticks were subsequently cleaned and stored in separate glass vials containing 75% ethanol. All tick specimens of *R. sanguineus* were identified to the species level on the basis of their morphological characteristics [16], and the external features of the *R. sanguineus* ticks were recorded using a stereo-microscope (SMZ 1500, Nikon, Tokyo, Japan) equipped with a fiber lamp and photographed for species identification. The genetic identity was also verified according to the mitochondrial 16S rRNA gene, as described previously [12].

### 2.2. DNA Extraction from Tick Specimens

Total genomic DNA was extracted from individual tick specimens used in this study. Briefly, tick specimens were cleaned by sonication for 3–5 min in 75% ethanol solution and then washed twice in sterile distilled water. Then, the individual tick specimen was immersed in a microcentrifuge tube filled with 180 μL lysing buffer solution supplied by the DNeasy Blood & Tissue Kit (catalogue no. 69506, Qiagen, Taipei, Taiwan) and homogenized with a TissueLyser II apparatus (catalogue no. 85300, Qiagen, Germany), as instructed by the manufacturer. The homogenate was centrifuged at room temperature, and the supernatant fluid was further processed by the DNeasy Blood & Tissue Kit, as instructed by the manufacturer. After filtration with the kit, the filtrated fluid was collected for quantifying the DNA concentration with a spectrophotometer (Epoch, Biotek, Winooski, VT, USA), and the extracted DNA was stored at −80 °C for further investigations [15].

### 2.3. DNA Amplification by Nested Polymerase Chain Reaction

Extracted DNA samples from each tick specimen were used as a template for PCR amplification. Two primer sets targeting the 16S rRNA gene were used for amplification. Initially, the primer set ECC (5′-AGAACGAACGCTGGCGGCAAGC-3′) and ECB (5′-CGTATTACCGCGGCTGCTGGCA-3′) was used to amplify all *Ehrlichia* spp., which produced a PCR product approximately 450 bp [18]). Then, nested PCR was performed using the species-specific primer set ECAN5 (5′-CAATTATTTATAGCCTCTGGCTATAGGA-3′) and HE3-R (5′-TATAGGTACCGTCATTATCTTCCCTAT-3′) for *E. canis*-specific amplification, which produced an amplicon of approximately 390 bp [18,19]. All PCR reagents and Taq polymerase were obtained and used as recommended by the supplier (Takara Shuzo Co., Ltd., Kyoto, Japan). Briefly, each 25 μL reaction mixture contained 1.5 μL of forward and reverse primers, 2.5 μL of 10× PCR buffer (Mg^2+^), 2 μL of dNTP mixture (10 mM each), 1 unit of Taq DNA polymerase, 3 μL of DNA template and was filled up with an adequate volume of ddH_2_O. In contrast, an adequate amount of sterile distilled water was added to serve as a negative control. PCR amplification was performed with a thermocycler (Veriti, Applied Biosystems, Taipei, Taiwan) and was denatured at 94 °C for 3 min, amplified for 35 cycles with the conditions of denaturation at 94 °C for 1 min, annealing at 55 °C for 2 min, and extension at 72 °C for 2 min. For the nested PCR, the following conditions were used: denaturation at 94 °C for 1 min and then amplified for 35 cycles with the conditions of denaturation at 94 °C for 1 min, annealing at 55 °C for 2 min and extension at 72 °C for 90 s, followed by a final extension step at 72 °C for 5 min.

All amplified PCR products were electrophoresed on 1.5% agarose gels in Tris-Borate-EDTA (TBE) buffer and visualized under ultraviolet (UV) light after staining with ethidium bromide. A 100-bp DNA ladder (GeneRuler, Thermo Scientific, Taipei City, Taiwan) was used as the standard marker for comparison. A negative control of distilled water was included in parallel with each PCR amplification.

### 2.4. Gene Sequencing and Phylogenetic Analysis

In general, 10 μL of each selected sample with a clear band on the agarose gel was submitted for sequencing (Mission Biotech Co., Ltd., Taipei City, Taiwan). The sequencing reaction was performed with 25 cycles under the same conditions and the same primer set of nested amplification by the dye-deoxy terminator reaction method using the Big Dye Terminator Cycle Sequencing Kit in an ABI Prism 377-96 DNA Sequencer (Applied Biosystems, Foster City, CA, USA). The resulting sequences were initially edited by BioEdit software (V5.3) and aligned with the CLUSTAL W software (Version 2.0) [27]. Thereafter, the aligned sequences of *Ehrlichia* gene from 11 Taiwanese strains were analyzed by comparing them with other *Ehrlichia* and outgroup strains (*Anaplasma phagocytophilum* and *Rickettsia rickettsii*) identified from various geographical and biological origins that are documented in GenBank (Table 1). Phylogenetic analysis based on the 16S rRNA genes was performed to indicate the genetic relationships among 24 strains of *Ehrlichia* and 2 outgroup strains (*A. phagocytophilum* and *R. rickettsii*) analyzed in this study. Phylogenetic trees constructed by maximum likelihood (ML) and neighbour-joining (NJ) methods were performed to estimate the phylogeny of the entire alignment using the MEGA X software package [28]. The genetic distance values of intra- and interspecies variations were also analyzed by the Kimura two-parameter model [29]. All phylogenetic trees were constructed and performed with 1000 bootstrap replications to evaluate the reliability of the construction, as described previously [30].

### 2.5. Nucleotide Sequence Accession Numbers

In this study, the nucleotide sequences of the PCR-amplified 16S rRNA gene of 11 *E. canis* from *R. sanguineus* ticks of Taiwan were registered with GenBank and assigned the following accession numbers: 97-TP-SL-07-sd06-M4 (OP389160), 97-TP-SL-09-sd08-M4 (OP389165), 98-TP-SL-06-sl02-EN3 (OP389212), 98-TP-SL-06-sl02-EN5 (OP389213), 97-TP-WH-08-sd06-M10 (OP392572), 97-TP-WH-08-sd06-M19 (OP392573), 97-TP-WH-08-sd06-M1 (OP392574), 97-TP-WH-08-06-F1 (OP392575), 97-TP-WH-08-sd06-M5 (OP392578), 98-TP-XY-03-sd01-M9 (OP392580) and 98-TP-SL-11-sd06-M15 (OP392581). For phylogenetic analysis, the nucleotide sequences of 16S rRNA genes from other 13 *Ehrlichia* strains and 2 outgroup strains were included for comparison, and their GenBank accession numbers are shown in Table 1.

## 3. Results

### 3.1. Detection of E. canis Infection in R. sanguineus Ticks of Taiwan

**Figure 1:**
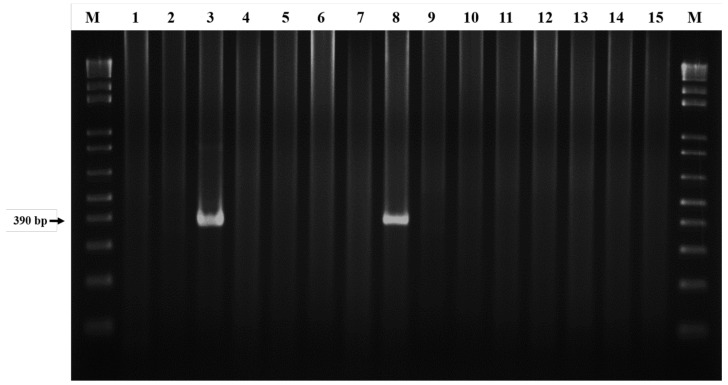
Molecular detection of *E. canis* infection in *Rhipicephalus sanguineus* ticks infesting dogs of Taiwan by a nested PCR assay targeting the 16S rRNA gene. M, 100 bp DNA marker; 1–14, sample numbers; 15, negative control. The expected PCR product is 390 bp for the 16S rRNA gene.

**Figure 2:**
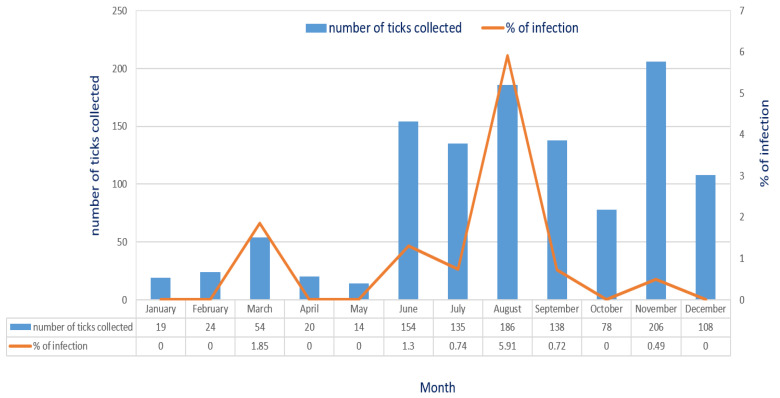
Monthly prevalence of *E. canis* infection and the number of *R. sanguineus* ticks collected from dogs in northern Taiwan.

Molecular detection of *E. canis* in *R. sanguineus* ticks was conducted using a nested PCR assay targeting the 16S rRNA gene (Figure 1). In general, a total of 1.42% (17/1195) of *R. sanguineus* ticks were detected with *E. canis* infection. Based on the life stage of ticks, *Ehrlichia canis* infection was detected in nymphs, males and females of *R. sanguineus* ticks with an infection rates of 0.60% (2/331), 1.31% (8/610) and 2.76%, (7/254), respectively (Table 2). The highest monthly prevalence of *E. canis* infection was observed in August with an infection rate of 5.91%, followed by the months of March, June, July, September and November with infection rates of 1.85%, 1.3%, 0.74%, 0.72% and 0.49%, respectively (Figure 2).

### 3.2. Genetic Analysis of E. canis Detected in R. sanguineus Ticks

To clarify the genetic identity of *Ehrlichia* in *R. sanguineus* ticks of Taiwan, the sequences of 16S rRNA gene fragments from eleven Taiwan *Ehrlichia* strains analyzed in this study were compared with the downloaded sequences of thirteen other *Ehrlichia* strains and two outgroup strains from different geographical and biological origins documented in GenBank. Results reveal that all these *Ehrlichia* strains detected in *R. sanguineus* ticks of Taiwan were genetically affiliated with the genospecies *E. canis* with a high sequence similarity of 99.7–100% and that can be clearly discriminated from other genospecies of *Ehrlichia* and outgroup strains (Table 3). In addition, intra- and interspecies analysis based on the genetic distance (GD) values of the 16S rRNA gene indicated a lower level of genetic variation (GD < 0.003 for *E. canis*) within the *E. canis* strains and a high level of genetic variation from other *Ehrlichia* spp. (GD > 0.009) and outgroup species (GD > 0.054) (Table 3).

### 3.3. Phylogenetic Analysis of Ehrlichia Strains Detected in R. sanguineus Ticks of Taiwan

**Figure 3:**
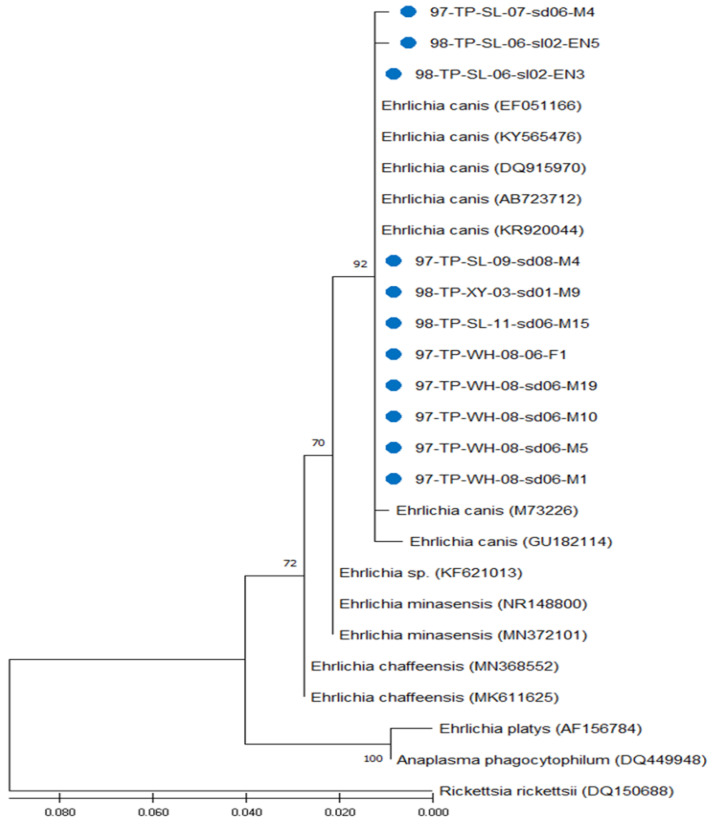
Phylogenetic tree based on the 16S rRNA gene. The aligned sequences of eleven Taiwan strains (indicated as ●) detected in *R. sanguineus* ticks of Taiwan were compared with available sequences from GenBank, including thirteen strains of *Ehrlichia* spp. and two outgroup strains identified from different biological and geographical origins. The constructed tree was analyzed by the Maximum Likelihood (ML) method using 1000 bootstrap replicates. Branch length is drawn proportional to the estimated sequence divergence. Numbers at the nodes indicate the percentage reliability of the tree.

**Figure 4:**
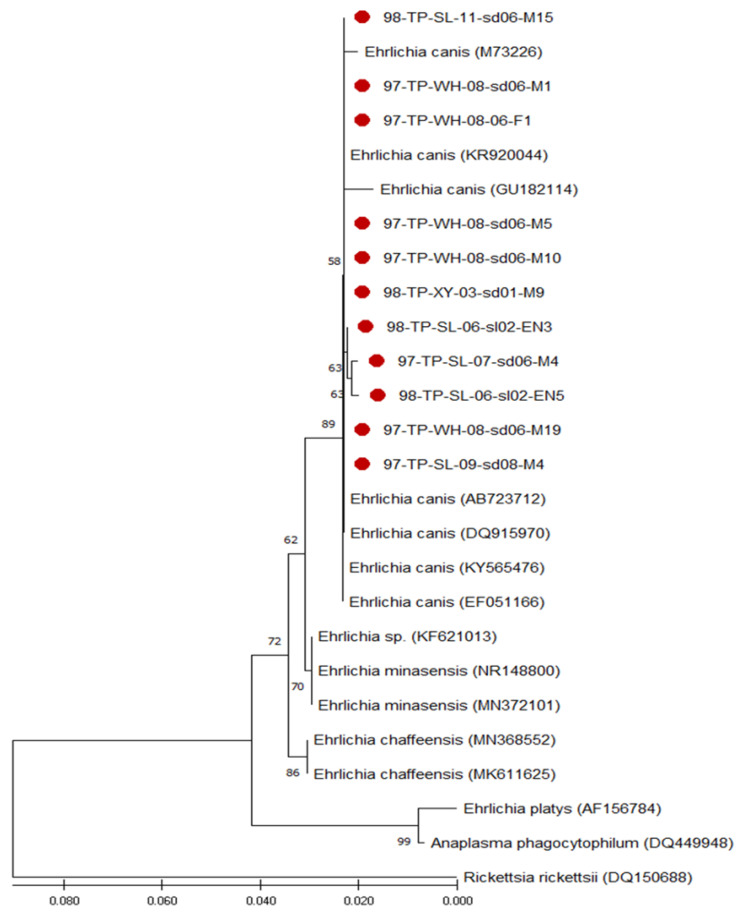
Phylogenetic tree based on the 16S rRNA gene. The aligned sequences of eleven Taiwan strains (indicated as ●) detected in *R. sanguineus* ticks of Taiwan were compared with available sequences from GenBank, including thirteen strains of *Ehrlichia* spp. and two outgroup strains identified from different biological and geographical origins. The constructed tree was analyzed by the neighbor-joining (NJ) method using 1000 bootstrap replicates. Branch length is drawn proportional to the estimated sequence divergence. Numbers at the nodes indicate the percentage reliability of the tree.

Bootstrap analysis was used to analyze the repeatability of the clustering of specimens represented in phylogenetic trees. Results indicated congruent basal topologies with three major clades of *Ehrlichia* that can be easily distinguished by ML analysis (Figure 3) and were congruent by NJ analysis (Figure 4). In general, all these *Ehrlichia* strains from Taiwan constitute a phylogenetic clade closely affiliated with the genospecies *E. canis* and can be discriminated from other *Ehrlichia* and outgroup species.

## 4. Discussion

This study provides the first molecular screening and genetic identification of *E. canis* in *R. sanguineus* ticks of Taiwan. In previous studies, *E. canis* was first identified as the causative agent of canine ehrlichiosis [3], and the first suspected human case was identified in asymptomatic patients from Venezuela [31]. Afterwards, *E. canis* was detected in the blood of patients with clinical signs suggestive of human ehrlichiosis [32]. Moreover, the comparison of ehrlichia 16S rRNA gene sequences revealed that the sequence profiles from the patients were identical to those from naturally infected dogs and *R. sanguineus* ticks [33]. In this study, *Ehrlichia* species detected in *R. sanguineus* ticks from Taiwan are genetically affiliated with the genospecies of *E. canis* with high sequence similarity (99.7–100%) to various *E. canis* strains identified from different biological and geographical origins (Table 3). Thus, our study provides the first molecular evidence and confirms sequences based on 16S rRNA genes of *E. canis* detected in *R. sanguineus* ticks of Taiwan.

The prevalence of *E. canis* infection in *R. sanguineus* ticks infesting dogs of Taiwan needs to be further investigated. In this study, the highest monthly prevalence of *E. canis* infection was observed in August (5.91%), and a higher prevalence was detected in early spring (March) and the summer season (June to August) (Figure 2). These observations are consistent with the zootiological survey of the seasonal abundance of tick populations infesting dogs described in our previous study [34]. Indeed, the warm climate from early Spring (March) to the hot summer season may enhance the searching activity of *R. sanguineus* ticks for feeding on dogs in the natural environment [35]. Indeed, the previous study also described that the higher prevalence of *A. platys* infection in dogs was observed in a heavily tick-infested kennel [36]. Thus, the seasonal abundance of *R. sanguineus* ticks is highly associated with the prevalence of *E. canis* infection in vector ticks and the geographical survey for the seasonal prevalence of *E. canis* infection in *R. sanguineus* ticks is essential for estimating the potential risk for human infection in Taiwan. Because of the close contact of dogs with humans, these observations demonstrate the importance of dogs serving as carrier hosts and highlight the potential risk for *E. canis* transmission to human populations.

Phylogenetic relationships among *Ehrlichia* spp. detected in *R. sanguineus* ticks can be determined by analyzing the sequence homogeneity of the 16S rRNA genes of *Ehrlichia* strains. Indeed, sequence analysis based on the 16S rRNA genes of *Ehrlichia* strains identified from different biological and geographical origins has been shown to be useful for evaluating the genetic relatedness of *Ehrlichia* strains among various hosts and tick species [18,19,20,21,22,23,24,25,26,32,33]. In this study, the phylogenetic analysis based on the 16S rRNA gene sequences of *Ehrlichia* strains detected in *R. sanguineus* ticks of Taiwan reveals identical similarity (100%) within these Taiwan strains and high genetic homogenity (99.7–100% similarity) affiliated with the genospecies of *E. canis* (Figure 3 and Figure 4). Indeed, phylogenetic analysis of this study also demonstrates that the genetic relatedness of *E. canis* detected in *R. sanguineus* ticks of Taiwan is mainly affiliated with the *E. canis* strains identified from dog blood in Malaysia, India, Taiwan and Portugal, respectively (GenBank accession no. KR920044, GU182114, KY565476 and EF051166) (Table 1, Figure 3 and Figure 4). In addition, genetic analysis based on 16S rRNA gene also revealed the discrimination of *E. canis* from the clades composed of other *Ehrlichia* and outergroup pathogens (*A. phagocytophilum* and *R. rickettsii*). The phylogenetic trees constructed by either ML or NJ analysis strongly support its discrimination (Figure 3 and Figure 4). Thus, the genetic identities of *Ehrlichia* strains detected in *R. sanguineus* ticks of Taiwan were verified as a monophyletic group affiliated with the genospecies of *E. canis*.

Although the biological mechanism for the transmission of tick-borne *E. canis* in *R. sanguineus* ticks remains controversial, *R. sanguineus* has been recognized as a main vector tick for the transmission of *E. canis* [37,38,39]. In previous studies, only ticks exposed to *E. canis* during the immature stages have been reported to transstadially transmit *E. canis* between dogs [40,41,42], and intrastadial transmission of *E. canis* by male *R. sanguineus* ticks has been experimentally proved in the absence of female ticks [43]. The co-feeding mechanism may account for another possible mode of transmission by ticks feeding closely to another infected tick on the same host, which may enhance pathogen transmission from an infected tick to a new tick [44,45]. In addition, co-infection with other tick-borne pathogens (such as *Anaplasma* spp., *Ehrlichia* spp., *Babesia* spp. and *Rickettsia* spp.) in *R. sanguineus* ticks has been described [20,46], and human infections will occur through the infective bite of *R. sanguineus* ticks.

The warming climate may be linked to the expansion of the geographical distribution of vector ticks, which will facilitate the transmission of tick-borne pathogens. Indeed, a previous study discovered that the *I. ricinus* tick is reported to have spread into previously unidentified northern areas of Sweden, Finland and Norway [47,48], and the warmer weather may enhance the attack/feeding activity of *R. sanguineus* ticks to dogs/humans [35]. Thus, it is possible that *E. canis* within *R. sanguineus* ticks can be transmitted to humans. Thus, further investigations focused on the prevalence of *E. canis* infection in relation to the geographical distribution of *R. sanguineus* ticks in Taiwan may help to illustrate the risk for transmitting of *E. canis* infection in the human population of Taiwan. In addition, epidemiological surveys regarding tick-borne zoonotic diseases in Taiwan needs to be further explored.

## 5. Conclusions

This study provides the first molecular evidence of *E. canis* in *R. sanguineus* ticks infesting dogs in Taiwan. The genetic relatedness based on phylogenetic analyses of 16S rRNA genes reveals genetic affiliation with the genospecies of *E. canis*. Because dogs serving as companion animals to humans, the discovery of *E. canis* in *R. sanguineus* ticks may draw attention to the potential transmission of tick-borne *E. canis* to humans in Taiwan.

## Figures and Tables

**Table 1:** *Ehrlichia*, *Anaplasma* and *Rickettsia* strains used for phylogenetic analysis in this study.

Strain	Origin of Bacterial Strain		16S rRNA Gene Accession Number ^a^
	Biological	Geographic	
Taiwan strain			
97-TP-SL-07-sd06-M4	*Rhipicephalus sanguineus*	Taiwan	**OP389160**
97-TP-SL-09-sd08-M4	*Rhipicephalus sanguineus*	Taiwan	**OP389165**
98-TP-SL-06-sl02-EN3	*Rhipicephalus sanguineus*	Taiwan	**OP389212**
98-TP-SL-06-sl02-EN5	*Rhipicephalus sanguineus*	Taiwan	**OP389213**
97-TP-WH-08-sd06-M10	*Rhipicephalus sanguineus*	Taiwan	**OP392572**
97-TP-WH-08-sd06-M19	*Rhipicephalus sanguineus*	Taiwan	**OP392573**
97-TP-WH-08-sd06-M1	*Rhipicephalus sanguineus*	Taiwan	**OP392574**
97-TP-WH-08-06-F1	*Rhipicephalus sanguineus*	Taiwan	**OP392575**
97-TP-WH-08-sd06-M5	*Rhipicephalus sanguineus*	Taiwan	**OP392578**
98-TP-XY-03-sd01-M9	*Rhipicephalus sanguineus*	Taiwan	**OP392580**
98-TP-SL-11-sd06-M15	*Rhipicephalus sanguineus*	Taiwan	**OP392581**
*Ehrlichia canis*	Unknown	USA	M73226
*Ehrlichia canis*	Dog blood	Malaysia	KR920044
*Ehrlichia canis*	Dog blood	India	GU182114
*Ehrlichia canis*	Leopard cat	Japan	AB723712
*Ehrlichia canis*	Unknown	USA	DQ915970
*Ehrlichia canis*	Dog blood	Taiwan	KY565476
*Ehrlichia canis*	Dog	Portugal	EF051166
*Ehrlichia* sp.	Cattle blood	Brazil	KF621013
*Ehrlichia minasensis*	*Rhipicephalus microplus*	Czech Republic	NR148800
*Ehrlichia minasensis*	*Hyalomma* tick	Egypt	MN372101
*Ehrlichia chaffeensis*	*Hyalomma* tick	Egypt	MN368552
*Ehrlichia chaffeensis*	Deer blood	USA	MK611625
*Ehrlichia platys*	Unknown	China	AF156784
*Anaplasma phagocytophilum* *Rickettsia rickettsii*	*Dermacentor silvarum* Dog	China USA	DQ449948 DQ150688

**^a^** Bold GenBank accession numbers were submitted by this study.

**Table 2:** Molecular detection of *Ehrlichia canis* in various life stages of *Rhipicephalus sanguineus* ticks parasitizing dogs by nested PCR assay targeting the 16S ribosomal RNA gene.

Life Stage of Tick	*E. canis* Infection Detected by Nested PCR		% of *E. canis* Infection
	Number of Ticks Positive	Number of Ticks Examined	
Nymph	2	331	0.60
Male	8	610	1.31
Female	7	254	2.76
Total	17	1195	1.42

**Table 3:** Intra- and intergroup analysis of genetic distance values ^a^ based on the 16S rRNA gene sequences between the *Ehrlichia* strains of Taiwan and other *Ehrlichia*, *Anaplasma* and *Rickettsia* strains documented in GenBank.

Bacterial Strains ^b^	1	2	3	4	5	6	7	8	9	10	11	12	13	14	15	16	17
1. 97-TP-WH-08-sd06-M1 (Taiwan)	–																
2. 97-TP-WH-08-sd06-M5 (Taiwan)	0.000	–															
3. 97-TP-WH-08-sd06-M10 (Taiwan)	0.000	0.000	–														
4. 97-TP-WH-08-sd06-M19 (Taiwan)	0.000	0.000	0.000	–													
5. 97-TP-WH-08-06-F1 (Taiwan)	0.000	0.000	0.000	0.000	–												
6. 98-TP-SL-11-sd06-M15 (Taiwan)	0.000	0.000	0.000	0.000	0.000	–											
7. 98-TP-XY-03-sd01-M9 (Taiwan)	0.000	0.000	0.000	0.000	0.000	0.000	–										
8. 97-TP-SL-09-sd08-M4 (Taiwan)	0.000	0.000	0.000	0.000	0.000	0.000	0.000	–									
9. 98-TP-SL-06-sl02-EN3 (Taiwan)	0.000	0.000	0.000	0.000	0.000	0.000	0.000	0.000	–								
10. *Ehrlichia canis*, Malaysia (KR920044)	0.000	0.000	0.000	0.000	0.000	0.000	0.000	0.000	0.000	–							
11. *Ehrlichia canis*, Japan (AB723712)	0.000	0.000	0.000	0.000	0.000	0.000	0.000	0.000	0.000	0.000	–						
12. *Ehrlichia canis*, Portugal (EF051166)	0.000	0.000	0.000	0.000	0.000	0.000	0.000	0.000	0.000	0.000	0.000	–					
13. *Ehrlichia canis*, USA (M73226)	0.003	0.003	0.003	0.003	0.003	0.003	0.003	0.003	0.003	0.003	0.003	0.003	–				
14. *Ehrlichia minasensis* (MN372101)	0.009	0.009	0.009	0.009	0.009	0.009	0.009	0.009	0.009	0.009	0.009	0.010	0.012	–			
15. *Ehrlichia chafeensis* (MN368552)	0.015	0.015	0.015	0.015	0.015	0.015	0.015	0.015	0.015	0.015	0.015	0.016	0.018	0.006	–		
16. *Anaplasma phagocytophilum* (DQ449948)	0.054	0.054	0.054	0.054	0.054	0.054	0.054	0.054	0.054	0.054	0.054	0.053	0.057	0.050	0.044	–	
17. *Rickettsia rickettsii* (DQ150688)	0.157	0.157	0.157	0.157	0.157	0.157	0.157	0.157	0.159	0.157	0.157	0.153	0.161	0.153	0.153	0.172	–

^a^ The pairwise distance calculation was performed by the Kimura two-parameter method, as implemented in MEGA X [28]. ^b^ Strains 10–15, 16 and 17 are the *Ehrlichia*, *Anaplasma* and *Rickettsia* strains documented in GenBank, respectively.

## Data Availability

All data generated and analyzed in this study are included in this manuscript, and all submitted GenBank sequences will be available from GenBank after publication.
